# Supplementary material for: Prone Positioning Decreases Inhomogeneity and Improves Dorsal Compliance in Invasively Ventilated Spontaneously Breathing COVID-19 Patients—A Study Using Electrical Impedance Tomography
Source: Diagnostics (Basel). 2022 Sep 21;12(10):2281. doi: 10.3390/diagnostics12102281 (PMC9600133; doi:10.3390/diagnostics12102281)
Supplement: Supplementary file 1 [file diagnostics-12-02281-s001.zip › diagnostics-1916898-supplementary.pdf]

diagnostics

MDPI

Prone Positioning Decreases Inhomogeneity and Improves Dorsal Compliance in Invasively Ventilated Spontaneously Breathing COVID-19 Patients—A Study Using Electrical Impedance Tomography

Supplementary Materials

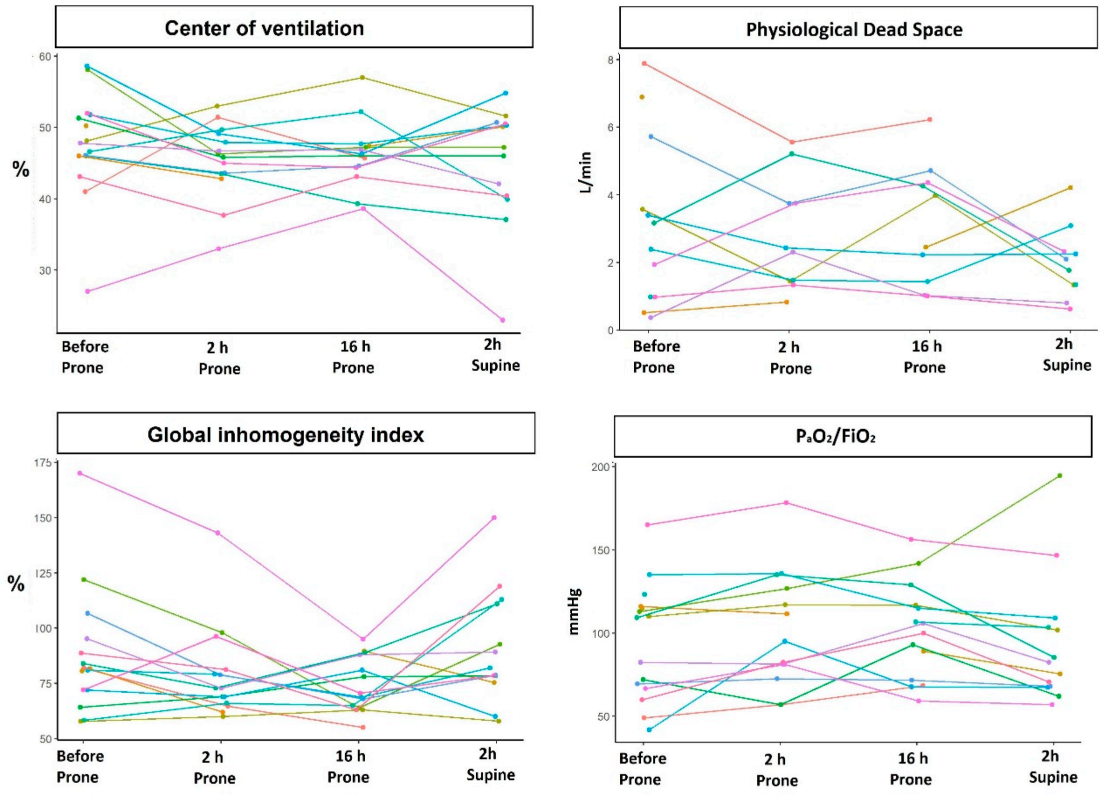

Figure S1. The dynamic effect of prone position (PP) on global ventilatory parameters.

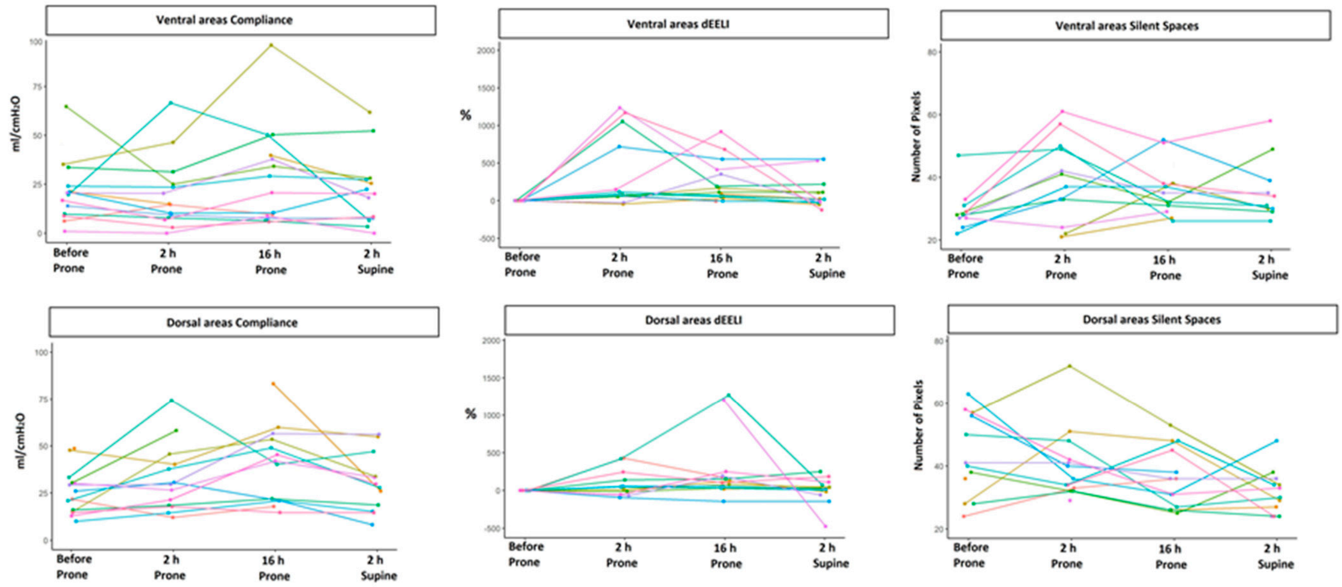

Figure S2. The dynamic effect of prone position (PP) on local compliance, changes in end-expiratory lung impedance, and silent spaces.
